# New York Children's Aid Society

**Published:** 1892-04-23

**Authors:** 


### New York Children's Aid Society.

New York, and indeed the United States in general, owe a deep debt of gratitude to Charles Loring Brace, founder of the Children's Aid Society. He was a philanthropist of the real sort—not a fussy busy-body who must have a finger in every pie, nor yet one of those self-advertisers who choose philanthropy for their special "line," and take care that they and their work shall be always *en évidence*, but a thoughtful, large-minded, loving-hearted man, who throughout his life devoted himself to the succour of the trodden down and oppressed, and, like a true humanitarian and Christian, sought for ways in which they might be helped to help themselves. In early manhood, having gone abroad for purposes of observation and study, he visited Hungary, and having been heard to express strong opinions anent the oppression of the people by the Austrians, he was clapped into gaol, and confined there for a month as a conspirator, only obtaining his release by communicating with the American Minister in Vienna. On his return to the United States he took up missionary work in addition to his professional labours as a journalist; and it was during this period that he became impressed with the terrible need for some society which should "save the children." To use his own words, "Our rather desultory and despairing labours for the reform of adult prisoners on Blackwell's Island, and the squalid poor in the Five Points district, proved a Sisyphus-like work, and soon discouraged all engaged in it. We seemed in those infernal regions to . . . fill the leaky vessel of Society by efforts which left it as empty as before. What soon struck us was the immense number of boys and girls floating and drifting about our streets with hardly any assignable home or occupation, who continually swelled the multitude of criminals, prostitutes, and vagrants."

The end of it was that he set on foot the New York Children's Aid Society, which, beginning in quite a small way, and having, as was once said, for its whole invested property an office-desk and chairs, has now, after nearly forty years of hard work, attained to grand proportions, having some twenty industrial schools in full work, as well as night-schools, lodging-houses, reading-rooms, summer-homes, and last, but not least, a Sick Children's Mission. Evidence of the need for such a society, and of the excellence of its work, may be found in the fact that since its inauguration similar societies have sprung up in Boston, Chicago, Brooklyn, and many other cities. The number of children reached by these agencies is now, therefore, very large.

It is hardly possible to over-estimate the importance of such efforts to reach, and to influence for good, the young. Sad experience teaches other workers, as well as Mr. Brace, that as the twig is, so will be the tree, and that efforts to reform a man, when he has grown up in evil ways, are—except in a few isolated cases—well-nigh hopeless. For the sake of the world in general, as well as for that of the lowest classes—the "dangerous classes," as Mr. Brace well called them—we must not grudge money and time spent upon the children. Indeed, to spend upon them is really the strictest economy, as who can doubt that knows the immense amount of money which our criminals annually cost us, to say nothing of the sums spent on precautions which their presence renders necessary?

It is surely a significant fact that, since 1859, when the Children's Aid Society may be said to have attained to strong working power, carefully collected statistics show a marked decrease in criminal vagrancy, especially among young people, although the population of New York has increased by many hundreds of thousands. It is very gratifying, too, to see that the number of children dying under five years of age from diarrhœal diseases, is lower than in former years, in spite of the increase in population; and doubtless this diminution is in part attributable to the work of the Sick Children's Mission, whose ten experienced doctors go from house to house through all the poorer quarters of the City, giving medical aid to neglected and suffering little ones, and sometimes to the mothers also, and reporting cases to the Mission in which better nourishment is urgently needed, and cannot be afforded by the parents themselves. The Boys' Lodging Houses speak for themselves, and also the Summer Home, where in 1890 over forty thousand children from the crowded tenement houses were entertained, some for merely a day, others for a week. To the Health Home come "tired mothers with their sick infants"; and a New York city missionary writes, "Of all the charitable deeds done by the people of New York, I think this one for the mothers and children the very noblest."

As for the Industrial Schools, those who know the almost insuperable difficulties connected with the enforcement of regular attendance at school of the children of our very lowest classes, can well understand that there is room for special schools for such children in New York, with its terrible over-crowding and mixed population. Here every effort is made to raise children "whose unfortunate condition, unattractive personal appearance, and uncertain attendance, make their presence unwelcome in the public schools, and thus virtually exclude them." Cooking, sewing, carpentry, and kindergarten work are taught, besides ordinary school lessons; in some of the schools dinners are given; and sometimes prizes of shoes, clothing, and firewood are offered to those who do well and attend regularly.

We must not omit to mention the very successful emigration work, over seventy-five thousand children having been placed in good homes in the West and South, and adopted by the farmers as their own. Very interesting letters are sent to the Secretary by these young emigrants, telling not only of their happy homes and new parents, but of the success of ventures made in later life by themselves. Says one young settler, after making out a list of his possessions—brood hogs, chickens, flour, and what-not—"You see I am pretty well prepared for this year's living, and why shouldn't I feel happy? And the best of all is I have a wife to live, and enjoy all of this, with me. If you wish, you have this read out to the boys of my old lodging-house, and if they don't believe it, as I used to be when I heard letters from boys read before I came West" (a wide-awake young rascal this!), "you tell them to write to me!"

Since the inauguration of the Children's Aid Society there has been but one branch of it which failed (and was speedily dropped)—the Workshops; and when its noble founder, worn out, it is to be feared, with his unceasing labours, lay down to die, far away from his native land, at St. Moritz, whither he had gone in hopes of restoring his failing health, that "consciousness of working with Christ towards the building up of His kingdom" which he had once spoken of as the highest reward of labour, must indeed have been his. He leaves many friends to mourn his loss, not only in America, but on this side of the water. One of his latest works, "Gesta Christi," will make him known to many Englishmen who never knew him in the flesh; but the best of all memorials will be found in the happy and self-respecting lives which, through his instrumentality, and by the grace of God, many thousands of his countrymen have been, and are now being, enabled to lead. When one thinks of the life and work of such men as the late Lord Shaftesbury and Mr. Loring Brace one is more than astonished that thousands of rich people would rather die of *ennui* than put their hands to philanthropic work of any kind.

